# Analysis of Communication, Team Situational Awareness, and Feedback in a Three-Person Intelligent Team Tutoring System

**DOI:** 10.3389/fpsyg.2021.553015

**Published:** 2021-03-01

**Authors:** Kaitlyn M. Ouverson, Alec G. Ostrander, Jamiahus Walton, Adam Kohl, Stephen B. Gilbert, Michael C. Dorneich, Eliot Winer, Anne M. Sinatra

**Affiliations:** ^1^Virtual Reality Applications Center, Iowa State University, Ames, IA, United States; ^2^U.S. Army Combat Capabilities Development Command Soldier Center, Natick, MA, United States

**Keywords:** situation awareness, team cognition, team communication, team training, intelligent team tutoring system, small group dynamics, intelligent tutoring

## Abstract

This research assessed how the performance and team skills of three-person teams working with an Intelligent Team Tutoring System (ITTS) on a virtual military surveillance task were affected by feedback privacy, participant role, task experience, prior team experience, and teammate familiarity. Previous work in Intelligent Tutoring Systems (ITSs) has focused on outcomes for task skill training for individual learners. As research extends into intelligent tutoring for teams, both task skills and team skills are necessary for good team performance. This work includes a brief review of previous research on ITTSs, feedback, teams, and teamwork, including the recounting of two categories of a framework of teamwork performance, Communication and Cognition, which are relevant to the present study. This research examines the effects of an intelligent agent, as well as features of the team, its members, and the task being undertaken, on team communication (measured by relevant key-presses) and team situation awareness (as measured by scores on a quiz). Thirty-seven teams of three participants, each at their own computer running a multiplayer surveillance simulation, were given just-in-time private (individually delivered) or public (team-delivered) performance feedback during four 5-min trials. In the fourth trial, two of the three participants switched roles. Feedback type, teamwork experience, and teammate familiarity had no statistically significant effect on communication or team situation awareness. However, higher levels of role experience and task experience showed significant and medium-sized effects on communication performance. Results, based on performance data and structured interview responses, also revealed areas of improvement in future feedback design and a potential benchmark for feedback frequency in an action-oriented serious game-based ITTS. Among the conclusions are six design objectives for future ITTSs, establishing a foundation for future research on designing effective ITTSs that train interpersonal skills to nascent teams.

## Introduction

Intelligent tutoring systems (ITSs) are computer-based instructional systems that interact with a single student to provide personalized instruction and one-to-one feedback as they progress through a learning experience. ITSs enable learning by assessing the current state of the student knowledge and skill mastery and by triggering adaptations of instructional content, timing, and teaching strategies (Wenger, 1987; Murray, 2003; Koedinger et al., 2013; Gilbert S. B. et al., 2015). ITSs have traditionally focused on the cognitive aspects of learning, such as assessing student content knowledge to trigger tutor feedback (Wood and Wood, 1999; Zakharov et al., 2008; Roll et al., 2011; Graesser et al., 2017). However, in the past decade, additional research has explored ITS learners’ affect and motivation (Mumm and Mutlu, 2011; Sabourin et al., 2011; Yang and Dorneich, 2016; Price et al., 2018), which are metacognitive factors not directly related to the task. More recently, efforts to create Intelligent Team Tutoring Systems (ITTSs) that offer automated coaching to teams expand this non-task focus to the challenge of how to tutor team skills, independent of a task. The present research evaluates the impact of an ITTS on the team skills of team situational awareness (SA) and communication.

Teams are defined as a group of two or more members, each with specific tasks that require coordination of information and activities to reach some common goal or objective (Salas et al., 1992). The coordination of tasks and information exchange are actions that may require some measure of training for a team to interact successfully. These interpersonal skills, such as emotional intelligence, cultural sensitivity, and communication, have been trained in individuals using distance learning, virtual practice environments, and ITSs (Lane et al., 2007; Riggio and Lee, 2007; Kumar et al., 2010; Orvis et al., 2010). While one-on-one training and systems encouraging the practice of interpersonal skills, or team skills, have shown promise, learning how to work within a team, individually, is only the first step. Training team skills should ideally happen in team settings with multiple learners interacting simultaneously within the same tutoring environment, such that the emergent and dynamic nature of the team task is well represented within the training.

Starting with systems that kept human trainers better apprised of the team’s whole performance (Zachary et al., 1999), work investigating ITTSs has branched from simple algorithms to social tutors giving team- and individual-level assessment (Kumar et al., 2010; Walton et al., 2015b; Ostrander et al., 2019). Within the environments of ITTSs, behavioral markers are used to identify team metrics (Salas et al., 2007b; Sottilare et al., 2018). From these metrics, ITTSs use feedback to present information about team and individual performance on task and team skills (Sinatra et al., 2018; Walton et al., 2018; Ostrander et al., 2019). Feedback from the tutor, in turn, influences teammates’ actions, interactions, and team SA. The present paper explores the implementation of ITTS feedback in a military-style simulated environment to guide the interdependent tasks of a team of three on both team and individual-level variables. For the team skills of communication and team SA, this research examined the impacts of feedback, environment experience, member role experience, and previous teamwork experience, and team-level teammate familiarity.

Before detailing the specifics of the environment, roles, and the intelligent team tutor, previous work in ITTSs, teams, and teamwork is reviewed. Additionally, two relevant categories of a framework of teamwork (Communication and Cognition) are reviewed. Finally, literature on feedback as it relates to team training is reviewed, and the hypotheses are presented.

### Previous ITTSs

Since the late 1990s, ITTSs have been developed to facilitate training on a variety of team tasks, including Naval air defense training (Zachary et al., 1999), mechanical engineering (Kumar et al., 2010), group coordination (Walton et al., 2015b), collaborative problem solving (Fiore et al., 2017), and team-coordinated surveillance (Ostrander et al., 2019). One of the first ITTS-like systems, the Advanced Embedded Training System (AETS), facilitated Naval air defense training (Zachary et al., 1999). The AETS monitored the learners’ button presses, speech, and eye movements to supplement a human trainer’s work. The human trainer’s time focused on aggregating data from the AETS into a team-level after-action review, while the AETS gave just-in-time automated task feedback to individual team members.

In the AETS, team members were assigned specific jobs, and feedback on performance was given by both the software agent and the human trainer. In the Team Multiple Errands Task (TMET; Walton et al., 2015b), the software agent, or tutor, supplied real-time individual and team-level feedback to a team of three as they completed a multiplayer virtual shopping task. The TMET extended a classic single-person shopping-based cognitive task, which has been used in cognitive rehabilitation to understand a patient’s executive function during tasks resembling typical life errands, to a team of three (Morrison et al., 2013; Walton et al., 2015b).

The team member roles required by a team task often play an essential role in the team’s dynamics. In TMET, the team members’ roles were homogeneous, with no specific required job roles or background training. In education and the workforce, team members often play different roles. For example, software development teams may consist of designers, engineers, and user researchers who work together to ship new products. While homogeneity makes the study of a team simpler and more controllable, the team tutor’s ecological validity in such situations is decreased.

The TMET tutor was not embodied or personified, and all feedback was given as brief phrases or data visualizations based on individual performance or team scores. A different kind of team tutor, Avis (Kumar et al., 2010), gave feedback to a team through conversational dialogue. Avis acted as a guide for learning underlying concepts of mechanical engineering. While Avis could be considered an ITTS, the tutor did not provide feedback for the team as a whole; it instead attended to each learner’s conceptual understanding. Further, Avis’s use of teams and conversation was used to facilitate the learning of the material rather than the improvement of team skills. Without team-level feedback and a lack of focus on team skills, Avis can be more accurately referred to as a socially capable tutor.

The agents developed within the 2015 Program for International Student Assessment (PISA) (Fiore et al., 2017) engaged learners in conversation in much the same way as Avis. Instead of tutoring as a facilitator, the agent (and sometimes multiple agents with various skills) worked to solve problems collaboratively with the learner as a peer. In this way, the PISA 2015 agents share similar goals to agents in tutoring roles; however, tasks always involved only one human teammate, rather than a multiple-human team. While the PISA 2015 encouraged the use of soft skills in engaging conversations, the inclusion of only one human per team limits their applicability to non-agent teams of two or more. Because no human–human coordination was necessary to the completion of the tasks, the team performance being measured in these programs better represented human–agent teamwork, which limits the potential application of such findings to human–human interaction.

Unlike Avis and the PISA systems, the surveillance team tutor (STT; Ostrander et al., 2019), similarly to AETS (Zachary et al., 1999) and TMET (Walton et al., 2015b), contained a fast-paced, high-cognitive load psychomotor-performance task that required steady, focused attention and did not typically allow for conversational dialog. The STT was designed to train two-person teams on a military task using just-in-time feedback tailored to individual player actions. Two types of feedback were given to STT users: Team feedback and Individual feedback. In the STT, feedback was coordinated using the Generalized Intelligent Framework for Tutoring (GIFT; Sottilare et al., 2012).

Generalized intelligent framework for tutoring is an open-source suite of modular tools designed to support the creation of ITSs in any domain. Similarly to other automated tutoring platforms, GIFT contains a learner module that tracks skills acquired by the learner, a domain module that can be programmed to hold expertise about common errors with recommended guidance, a pedagogical module that recommends the form of instructional interventions, and a communication module for whatever user interface the learner is using. In a previous effort, the authors adapted GIFT to enable the development of an ITTS and used that architecture for the present study. More details on these efforts are described elsewhere (Gilbert S. et al., 2015; Gilbert et al., 2017; Brawner et al., 2018; Walton et al., 2018). In effect, the GIFT team approach enabled each team member to have an individual tutor and for there to be one tutor for the whole team. For a team of three, for example, GIFT would contain four tutors. The feedback received by an individual would be the combination of the individual’s tutor’s feedback and the team tutor’s feedback. This is certainly not the only feasible architecture for a team tutor, but it was a novel and unique approach at that time.

The military task for STT was developed in Virtual Battlespace 2, a serious game engine. In the task, two spotters were positioned on top of a building in the middle of a virtual village environment, which included walls as obstacles between which OPFOR (OPposing FORces) could run. Each spotter was responsible for watching a zone consisting of half of the environment and alerting their teammate to OPFOR who neared that teammate’s zone. The full task consisted of a *Transfer* event, in which one spotter alerted the other to an approaching OPFOR; an *Acknowledge* event, in which the receiving spotter acknowledged the transfer; and an *Identify* event, in which the second spotter noted receiving the OPFOR into their zone. Teammates passed this information to each other via a verbal communication channel and to the tutor using pre-assigned keyboard keys.

While the tutor was shown to have limited impact on the performance of participants and their teams (Ostrander et al., 2019), there were promising results related to the impact of feedback on shared mental models and overestimation of performance. Feedback on the Acknowledge subtask did result in fewer errors, and feedback given on team-level tasks, in general, reduced the tendency of individuals to rate their teammates as having performed poorly. Lastly, for the conditions in which participants received no feedback or individual-level feedback, participants’ self-ratings of individual performance did not correlate significantly with their tutor-assessed performance, while they did in the team-level feedback condition. These results suggest promise for team training with an ITTS. The ITTS developed for this paper was a three-person extension of the two-person STT and will be described in the section “Materials and Methods.”

### Teams and Teamwork

Teams have been a topic of study for nearly a century, starting with examinations of groups working together in factories and developing into a depth of work seeking to uncover the components that make up a good team (Bisbey et al., 2019). In 2007, 138 frameworks that described teamwork and team performance were identified (Salas et al., 2007b). Since then, researchers have highlighted markers of team performance that can help identify teamwork abilities as they occur, including in situations where outcomes are either not directly quantifiable or occur over an extended period, and synthesized research on team success metrics to standardize the terminology and direct future team research (Salas et al., 2009; Wiese et al., 2015). By standardizing the language surrounding components of teamwork, team success and teamwork ability can be defined.

As presented by Salas et al. (2015), team performance is influenced by several metrics. Of particular importance to the current research effort are the concepts of Communication and Cognition. Each will be discussed below, followed by another influence important to this specific context: Feedback.

#### Communication

Communication is central to teamwork and is recognized as a promising marker for team ability. Communication in the form of non-task conversation facilitates team members’ social connections with one another, or group affinity, and in turn, the team members gain the willingness to communicate with one another (Nardi, 2005). Team members may gain group affinity through this non-task conversation, or they may come in with pre-existing professional or personal relationships. Such familiarity has been shown to directly improve performance (Mason and Clauset, 2013) and increase a team’s effectiveness under high workload (Smith-Jentsch et al., 2015). In distributed teams, which interact through computer-mediated communication (CMC), there is a concern that the lack of natural opportunity for the non-task conversations that may occur during hallway passing, for example, will negatively impact team performance (Oren and Gilbert, 2011, 2012). One metric for a distributed team’s success may lie in the team’s ability to leverage benefits and minimize deficits of CMC technology (Pinjani and Palvia, 2013; Alsharo et al., 2017). To enable the development of group affinity and set distributed teams up for success, an intelligent tutor may be able to guide the use of such CMC technology.

In ITSs, the learner traditionally communicates with the intelligent tutor through their keyboard, responding to questions with typed statements (Graesser et al., 2017). However, ITTSs pose two challenges to this communication method: (1) learners must communicate with their teammates as well as the tutoring system, and (2) the tasks tend to be fast-paced, which inhibits the learner’s ability to type sentence-level responses. In ITTSs that use fast-paced tasks, such as the TMET (Walton et al., 2015b), game-state events are recorded as the learner interacts with the task and their teammates to facilitate feedback. Indeed, in the present ITTS, communication was recorded by the game (and tutor) as the learner pressed pre-defined keys to indicate when and what form of information was being shared. Communication performance in the present work is understood as the timeliness and quantity of communication recorded through single key-presses instead of full sentences.

#### Cognition

Team Cognition is defined as the group’s ability to function cooperatively toward a common goal, and it relies on the team’s awareness of the common team and shared situations, or their team SA (Salas et al., 2015). When team members actively contribute to their team Cognition, responsibility for teammate actions should be more fairly attributed, whether to the teammate’s personality or an external circumstance (Cramton et al., 2007). This team Cognition could be introduced by Communication, as discussed above, or by allowing team members to experience each other’s roles. Role switching, which establishes a grounded understanding of the team’s roles, can create a more functional environment in which teammates are able to anticipate member actions and cover extraneous responsibilities when necessary (Sottilare et al., 2011). By experiencing another team member’s role, one gains a personal understanding of that role’s requirements.

##### Team SA

Team SA is a product of both communication and cognition within a team. Team SA includes the awareness of teammates’ abilities, team-level tasks, and current goings-on in the situational context. Such awareness leads to a decrease in situational invisibility, meaning a teammate’s sudden inability to complete their tasks is more believably a cause of their situation rather than their skill level (Cramton et al., 2007), and a more effective team (Smith-Jentsch et al., 2015). Team SA, in this context, is a team-wide understanding of the shared goals and the tasks required to achieve them, as well as an awareness of each member’s environment.

As teams gain experience together, their team Cognition develops through the creation of shared mental models, which, in turn, decreases the need for task communication (Carpenter et al., 2008). Reagans et al. (2005) found that experience within a team, and with one’s role on that team, impacted team performance. Therefore, while Communication is necessary for developing group affinity and team Cognition, it may become less necessary as teams gain familiarity with their responsibilities and with one another.

In previous research, information sharing is also a recognized path to better team outcomes such as task success and creative solutions to problems (Alsharo et al., 2017). In teams, situational communication must occur between team members for team SA to spread; however, in a training scenario, the supervising agent or person could inject such information into the team’s Cognition through feedback. For this reason, the impact of feedback on team Cognition (by way of team SA) is evaluated in this study through an appraisal of each teammate’s knowledge of the team’s tasks and of the team’s shared mental model of the completed task.

#### Feedback

Proper feedback, when aimed at increasing awareness of the task or of the process, or at increasing self-regulation, has positive impacts on learning (Timperley and Hattie, 2007; Gilbert et al., 2017). Feedback can enable each team member to identify the goals of their respective roles within the team, in essence helping them to create a shared mental model of an “expert team.” While performance on task work has been shown to be most affected by privately given feedback, teamwork has been affected by publicly given feedback (Geister et al., 2006; Mumm and Mutlu, 2011), although feedback-effectiveness results in other studies have been mixed (Peñarroja et al., 2015; Ostrander et al., 2019).

Properly calibrated feedback must align with the expert model. It must also follow proper etiquette for the team context. Feedback that ignores etiquette alters team members’ willingness to welcome the help of a tutor (Dorneich et al., 2012; Walton et al., 2014), while excessive messages (Price et al., 2018) have been shown to harm learner performance in ITS situations. This can be solved through attention to the quantity of messages and to the affect portrayed by those messages. For example, by reducing the number of messages a tutor supplies during expected times of high workload, the tutor’s chance of interrupting learners is reduced, and thus etiquette norms are maintained. In the present study, feedback was implemented to teach the task and team skills required of participants for effective completion of the team task.

### Hypotheses

The researchers expect that feedback delivered to the whole team, here referred to as “public,” would have a positive effect on the team SA since the use of team-level feedback will result in more shared information than individualized feedback (Geister et al., 2006). Additionally, public feedback would allow teammates to keep track of their whole-team performance, correcting and encouraging others to correct as necessary. It was hypothesized that:

H1: Public feedback will result in higher team situational awareness than private feedback.

Changes in communication should happen naturally within the course of any team. Because feedback is provided to teams in this experiment, the communication performance should improve over time. A previous analysis of this study that focused on task performance revealed a decrease in self-reported mental workload over time (Ouverson et al., 2018), which is indicative of learning effects. If participants are learning as they participate longer in the study (e.g., complete more trials), it is expected that they will have more mental resources to contribute to communication and are likely learning the nuance of the task-required communication. Therefore, it is hypothesized that:

H2: Communication will improve as a participant gains experience in the task.

In the present study, two participants on each team switched roles in the last of four trials. Role switching establishes a grounded understanding of the team’s roles and has been shown to foster more effective communication in the long term (Sottilare et al., 2011). Therefore, it is hypothesized that:

H3: When stepping into a new role and having experience in the task (Trial 4), individual communication will be better than when a participant has neither role nor task experience (Trial 1).

The current paper also investigates how the amount of role experience influences team SA. Sætrevik and Eid (2014) note the importance of SA for each member of a team, stating that for a team to perform its best, members must understand their tasks within the team context. This understanding is developed through training and experience with the task and the team’s roles. It was hypothesized that:

H4: For the participants who experience two roles, team situational awareness will be higher than that of participants who experience only one role.

Communication is necessary for developing group affinity and team SA, but it may not be as necessary as team members gain familiarity with one another. However, the present study’s use of feedback to actively encourage team communication is expected to counter familiarity’s communication-reduction potential. An individual who knows at least one of their teammates should be more comfortable communicating within the team. It is therefore hypothesized that:

H5: Teams with members who have prior familiarity will have better communication than those whose members do not.

Lastly, previous experience in teams across domains should logically impact an individual’s future performance within a team. Research points toward the impacts of previous teamwork experience on future teamworking attitudes (Rudawska, 2017) and the understanding of how to perform well on future teams (Rentsch et al., 1994; Reagans et al., 2005; Hirsch and McKenna, 2008). The tie between team experience and performance has, thus far, been indirect; an individual’s experience has influenced his or her schema of teamwork, which in turn influences that person’s performance in a team setting. The researchers seek to test this connection directly, hypothesizing that:

H6: Persons who have greater experience working in teams will have higher team situational awareness.

Next, the study’s methods are presented, including a description of the task, the feedback mechanism, and the data analysis methods.

## Materials and Methods

### Participants

Participants (*N* = 111) self-identified as 45 females, 61 males, and five persons who did not self-identify or disclose their gender. The average age of the sample was 23.2 years of age (*SD* = 7.8). Nearly every participant (89%) reported working in teams at least once a month, and the majority (88%) reported enjoying teamwork. Seventy-two participants (65%) reported playing videogames; just over half (*M* = 55%, *SD* = 30%) of those video games involved teams or cooperative play.

The participants completed the experiment in teams of three (*N_*teams*_* = 37), which were determined during experiment sign-up. Participants could either be randomly assigned to teams or sign up with a group. As such, 36% of participants had met at least one person on their team before the experiment, yielding 17 teams (46%) with members with some familiarity and 20 (54%) teams whose assignment was fully random.

### Task Overview

The ITTS developed for this experiment was called the Targeter-Enhanced Surveillance Team Tutor (TESTT), and it was a three-person extension of the two-person STT (Ostrander et al., 2019). The goal in the TESTT was for a team of three to pass targets from one teammate to another as they moved from one side of the virtual environment to the other, ending with a threat assessment in which the level of potential threat posed by each target is reported to the tutor and the team. Each team contained two surveillance Spotters whose primary duties were transferring potential OPFOR, or opposing forces, to each other as they cross the two zone boundaries, designated as the one-pole boundary and two-pole boundary. The third member of their team, the Targeter, stood in a tower with a broad overview of both zones, examined potential OPFOR (entities), and used the keyboard to indicate which of three levels of threat each person posed.

**FIGURE 1:**
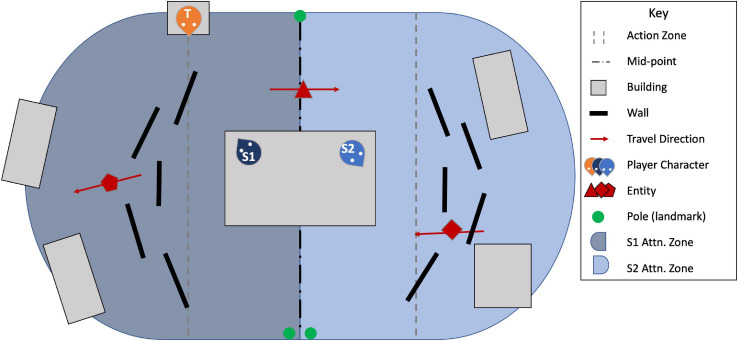
Top–down view of the simulation environment, with detail reduced for simplicity. The Targeter is marked with a T and stands on a building (top-left), while Spotter 1 (S1) and Spotter 2 (S2) share the central building and each scan their own attention zone. As the entities move through the action zone (bounded by the gray dashed-lines), players performed the five subtasks detailed above, with the hand-off from S1 to S2 occurring as the entities cross the mid-point. Adapted with permission from Ouverson et al. (2018).

It should be noted that during the experiment, participants referred to each other as “Spotters” and “Snipers.” In this paper, we use the term “Targeter” instead of “Sniper” to better reflect the role, which *targets* potential OPFOR and assesses their level of threat without ever engaging with a sniper rifle. Figure 1 shows a representative top–down view, with environment detail reduced for simplicity, and the full sequence of required subtasks in each trial is detailed in the following example:

S*potter 1 sees an entity in her attention zone approaching the action zone. “One at pole one,” she asserts, pressing the assigned key for pole one (“1”) to* **Transfer** *the entity to Spotter 2. “Okay,” says Spotter 2, striking the “E” key to* **Acknowledge** *the transfer. After the entity passes the midpoint, entering his attention zone, Spotter 2 alerts the Targeter, “There’s someone at pole 1, Targeter,” and presses the spacebar* (**Identify***). “Got it,” the Targeter* **Acknowledgments** *(again, using the “E” key), and uses the “B” key to zoom in on the entity and* **Assess** *the threat it poses. Seeing that the entity is a civilian, the Targeter keys “Z,” rather than “X” or “C,” which are used to signify an OPFOR wielding a gun but not wearing a vest (Level 1) and a vest-wearing OPFOR (Level 2), respectively.*

### Procedures

Upon receiving approval from the Institutional Review Board, participants were recruited using an all-student-and-staff mailing list at a large Midwestern university. Before they signed up for a timeslot, participants were required to give informed consent followed by basic demographic information. When they arrived, participants were randomly assigned roles. Each participant completed a familiarity survey, which asked whether they knew their fellow participants, and watched a 7.5-min tutorial video. The video introduced the task, the environment, and the controls for each role. A reference sheet with a small table for each role was included to aid in key assignment memory retrieval. Participants entered three separate rooms to use individual computers, but there was an open audio channel connecting the rooms.

Participants began the first of four 5-min trials after they confirmed they understood the study by answering the attending researcher’s verbal quiz on action timing and associated key-presses. In the fourth trial, two of the three teammates switched roles, and just before starting in the new configuration, all players were given a chance to ask questions about their role. Because of this role switch, a practice trial was not used to ensure that the fourth trial’s role newness was similar to that of Trial 1.

After each trial, the participants were asked to complete a post-trial survey. After the entire experiment, the participants were asked to complete a post-experimental survey and participate in a structured interview, led by an experimenter, with their teammates regarding the experimental environment and the feedback.

This paper uses *Explanatory variables* to refer to Independent and Quasi-independent variables and *Response variables* to refer to the Dependent variables. This choice reflects the use of linear mixed-effects models (LMMs) in the data analysis (see Futing Liao, 2011; Bates et al., 2015). The next sections detail these variables.

### Explanatory Variables

#### Feedback Privacy

For each experimental session, feedback privacy served as an explanatory variable with two between-subjects levels wherein either (1) feedback was shown only to the person to whom it applied (heretofore referred to as “private feedback”) or (2) all feedback was shown to everyone on the team (“public feedback”). Participants received feedback on their performance, provided to them in real time by an ITTS. Imagine this manipulation as a tutor individually contacting each learner about their performance (private), or the tutor telling everyone that an issue with an anonymous team member’s performance has surfaced, regardless of its relevance to each team member (public).

#### Trial

There were four trials per experimental session. This explanatory variable was used to examine how experience with the task influenced the response variables detailed below.

#### Participant Role and Role Pattern

In this experiment, participants were assigned to one of two kinds of roles: Targeter or Spotter. Two of the team members were Spotters, who watched the virtual environment and indicated via button press when a potential OPFOR crossed from one zone to the other. The other team member, the Targeter, used information given by the Spotters to locate and assess the threat level of the potential OPFOR. Two of the three participants switch roles in the fourth trial. This creates the explanatory variable of role pattern, with three levels: either Spotter in all four trials (SSSS), Spotter for the first three and Targeter in the last (SSST), or Targeter in the first three and Spotter in the last (TTTS).

#### Teamwork Experience

In a survey given prior to the start of the study, each participant’s teamwork experience frequency was recorded. Individuals self-reported the frequency with which they work in teams. While this was not a manipulated variable, this variable served as an explanatory variable with three levels: High, Moderate, and Low frequency. The levels correspond to participant responses, where High frequency corresponds to “daily,” Moderate frequency represents responses of “2–3 times a week” and “once a week,” and Low frequency maps to “2–3 times a month,” “once a month,” and “less than once a month.”

#### Teammate Familiarity

Participants assigned their own teams by registering for the study as random individuals or with friends, as described above. Thus, varying levels of prior teammate familiarity were observed and used as an explanatory variable. Before starting the study, a survey assessing baseline relationships within the teammates was given to each participant. For each teammate, the survey asked, “Have you met teammate X?” While teams could have partial or full familiarity (in addition to no familiarity), only differences between teams in which familiarity does not exist (none of the teammates indicated they had met another member; 19 teams) and those in which familiarity exists (at least one team member said they had met a teammate; 17 teams) were examined in this study.

### Response Variables

Response variables were derived from scores on quizzes given during the post-experiment survey and data collected by the tutor during the experiment, as shown in Table 1. The mean score associated with each response variable (Range: 0–100%) is also given in Table 1. Each of these variables is discussed below, and Tables 2, 3 show the items that make up these surveys.

**TABLE 1:** Response variables examined in this paper.

**Response variable**	**Metric**	**Frequency**	**Mean**	***SD***
Targeter Goal Awareness	Proportion correct on Targeter goal quiz (0–100%)	Post-experiment (1×)	79%	22%
Shared Role Awareness	Similarity to teammates on Targeter and Spotter goal quizzes (0–100%)	Post-experiment (1×)	72%	14%
Team Task Awareness	Spearman rank correlation of task quiz answers to correct answers (0–100%)	Post-experiment (1×)	70%	26%
Communication	Percentage of prompt acknowledges (0–100%)	Each trial (4×)	36%	27%

**TABLE 2:** Shared Role Awareness and Targeter Goal Awareness quiz items.

**What are the Goals of the Targeter in this Task?**	
□ To identify targets new to their zone	□ To keep count of how many targets have left and entered their zone
□ To identify targets leaving their zone	□ To keep count of how many OPFOR are on the map
□ To assess the treats posed by targets	□ To keep count of how many civilians are on the map
□ To acknowledge what their teammates say	□ To count the number of OPFOR wearing vests
**What are the Goals of the Spotters in this Task?**	
□ To assess the threats posed by targets	□ To keep count of how many civilians are on the map
□ To keep count of how many targets have left and entered their zone	□ To count the number of OPFOR wearing vests
□ To keep count of how many OPFOR are on the map	

**TABLE 3:** The task quiz answer key.

**Task steps**	**Order**
Spotter 1 sees a target approaching the 1 pole	1
Spotter 1 transfers a target by pressing the 1 key	2
*Spotter 1 transfers a target by pressing the E key*	*NA*
Spotter 2 acknowledges his/her teammate’s communication by pressing the E key	3
*Spotter 2 acknowledges his/her teammate’s communication by pressing the 1 key*	*NA*
Spotter 2 sees a target near the 1 pole	4
Spotter 2 identifies that a target has entered his/her zone by pressing the SPACEBAR key	5
*Spotter 2 identifies that a target has entered his/her zone by pressing the E key*	*NA*
*Spotter 2 identifies that a target has entered his/her zone by pressing the 1 key*	*NA*
Spotter 2 informs Targeter that a target has entered his/her zone	6
Targeter acknowledges his/her teammate’s communication by pressing the E key	7
Targeter searches for a target in Spotter 2’s zone in the direction of the 1 pole	8
Targeter spots a target and assesses the threat posed by the target	9
*Targeter believes target to be a civilian and presses the C key*	*NA*
*Targeter believes target to be a civilian and presses the X key*	*NA*
Targeter believes target to be a civilian and presses the Z key	10

*This key is a list of steps to the task and the order in which they should occur. Participants received these steps presented in random order and were asked to order them and mark certain ones as erroneous. “NA” and italics flag the erroneous steps in this answer key.*

After the fourth trial, a post-experiment survey was given. Shared Role Awareness, Targeter Goal Awareness, and Team Task Awareness were derived from answers to three quizzes (Targeter goals, Spotter goals, and task) in this post-survey as a measure of team SA. The inclusion of task and role quizzes (e.g., Targeter and Spotter goal quizzes) mirrors the efforts of Sætrevik and Eid (2014), who use expert accounts of teamwork to measure team SA. The quizzes in the present study were based on the tutorial video given to all participants at the beginning of the study.

Participants were given a list of actions (shown in Table 2) and were asked to identify which actions were goals of the Spotters in the task and the goals of the Targeter in the task. The similarity of each participant’s answers to their teammate’s answers for each question on both the Targeter and Spotter goal quizzes, calculated by Spearman Rank Correlation, made up the Shared Role Awareness score, while Targeter Goal Awareness was simply the score on the Targeter goal quiz. Participants, on average, scored 72% on the Shared Role Awareness score (*SD* = 14%) and 79% on the Targeter Goal Awareness score (*SD* = 22%).

Additionally, participants were given a list of statements of steps to the task for the task quiz (Table 3). They then sorted statements into two categories depending on whether they occurred in the task or not and were asked to order the steps of the task correctly. By finding the Spearman Rank-Order correlation of each participant’s answers, the Team Task Awareness score was derived. On average, participants scored 70% on this quiz (*SD* = 26%).

Communication was measured as the percentage of prompt acknowledges via an analysis of the participant’s keystrokes during the trials. This was chosen as the metric because while teams were instructed to use Acknowledgment at certain points in the action sequence, this action was not pivotal to the team’s end goal: assessing the threat posed by potential OPFOR in the environment. Therefore the Acknowledge action was similar to the secondary communication used to develop group affinity (Nardi, 2005) and served as a proxy measure of the communicativeness of the team.

The metric of prompt acknowledgment as a measure of communication is coarse; it does not explicate the message’s content, whether the information is understood, or whether the message is redundant and unnecessary. Because this metric is coarse, it would not be clear if more communication is better without task context. However, the present experiment begins with training participants on a protocol that requires a certain amount of communication. In this protocol, more acknowledgments *are* better (up to a pre-determined number corresponding to the number of entities in the scene), and communication timeliness can also be measured against the other players’ game events and actions. On average (and averaging over trial), a participant’s Acknowledgment percentage score was 36% (*SD* = 27%).

### Experimental Design

**FIGURE 2:**
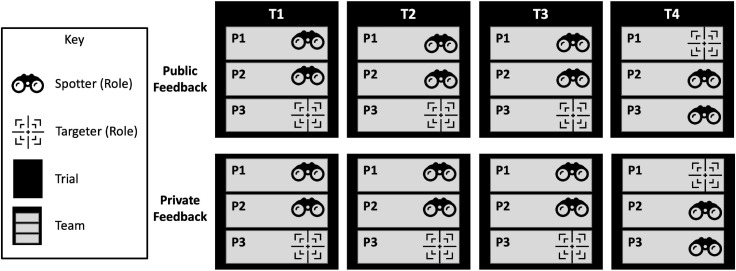
A visualization of the experimental design, showing that for each team (black group of gray rectangles) there were four trials in one of two feedback conditions. The three team members experienced the trials as Spotters or Targeters, or both.

The experiment followed a nested repeated measures design, in which each experimental session consisted of one team of three participants. The design is nested because the independence of individual participants cannot be assumed due to their organization into teams. Each team completed four trials with an ITTS that provided either Public or Private Feedback. As shown in Figure 2, two of the three participants on each team experience a role switch in Trial 4—in this case, P1 and P3 switch roles. The role switch was randomly assigned before the start of the experiment to happen to either P1 and P3 or P2 and P3, where P1 and P2 always start in the Spotter role.

Because of this unique experimental design, care was taken to ensure that data analysis methods were statistically valid. As such, only Trials 1 through 3 are considered when comparing communication performance data. Since two participants switched roles after Trial 3, Trial 4 data are only used when making comparisons related to role pattern.

#### Task Feedback

Full descriptions of the feedback delivery mechanism for the TESTT and other task feedback design considerations are described in Walton et al. (2014) and Gilbert et al. (2018), so only a brief recount of relevant information shall be given here.

The tutor gave feedback for the Transfer, Acknowledge, Identify, and Assessment subtasks by consulting the programmed conditions by which that feedback was to be triggered or not triggered. The type of feedback (either Private or Public) was determined by the tutoring paradigm, selected prior to the beginning of the procedure. Subtasks were evaluated based on their timeliness as either *Below Expectation*, *At Expectation*, or *Above Expectation*, categories given in army training (Goldberg and Hoffman, 2015). To avoid having feedback triggered too frequently (by every player action), feedback was triggered by multiple errors (either incorrect actions or missing actions) of the same type. More information is given in Gilbert et al. (2017) and Ostrander et al. (2019).

Examples of feedback given to the Spotters and Targeters are shown in Table 4. The Public feedback condition would have typically resulted in participants receiving more than twice the feedback as those in the Private condition since each teammate would have received feedback triggered by all three team members rather than only the feedback triggered by their own actions. To balance the amount of feedback received in the Public condition with the amount received in the Private condition, feedback was not given for Transfer events in the Public condition. Since the Acknowledge actions were directly tied to the Transfer or Identify actions, Acknowledge feedback was framed as being triggered by the Transfer–Acknowledge or Identify–Acknowledge pairs in the Public condition.

**TABLE 4:** Examples of feedback given for each player subtask.

**Task**	**Feedback**
Transfer	*“It is important to effectively communicate crossings”*
Acknowledge	*“It is important to confirm at appropriate times”*
Identify	*“It is important to identify targets as quickly as possible”*
Assessment	*“Remember to assess the threats posed by all crossing targets”*

*Feedback was given for errors after they occurred a pre-established number of times. Condition refers to the feedback level of the experiment in which the feedback was given.*

### Data Analysis

Hypothesis testing was done by fitting four LMMs using the restricted maximum likelihood (REML) criterion, which were generated in RStudio using the lme4 package (Bates et al., 2015). These LMMs are shown in Table 5. Estimated Marginal Means were calculated using the emmeans package (Lenth, 2019). This approach, rather than the standard ANOVA and its variants that assume independent data, was used to account for the fact that teammates cannot be considered independent of one another. Hypotheses 3 and 5 (H3 and H5, which are missing from the table) were tested using more traditional methods, which is explained later in this section.

**TABLE 5:** Linear mixed-effects models (LMMs) and the hypotheses tested using them.

**Linear mixed-effects model**	**Response variable level**	**Hypotheses tested**
Shared Team Awareness = Role pattern + Team experience frequency + Feedback privacy + Random effect(Team)	Individual	H1, H4, H6
Targeter Goal Awareness = Role pattern + Team experience frequency + Feedback privacy + Random effect(Team)	Individual	H1, H4, H6
Shared Role Awareness = Role pattern + Team experience frequency + Feedback privacy + Random effect(Team)	Individual	H1, H4, H6
Communication = Role pattern + Feedback privacy + Trial + Random effect(Team)	Trial	H2

The first three models expressed Shared Team Awareness, Targeter Goal Awareness, and Shared Role Awareness as a function of role pattern (SSSS, TTTS, and SSST), the level of self-reported team experience frequency (low, moderate, or high), feedback privacy (Public or Private), and a random effect of the team to which each participant belonged. These response variables were measured once after the experiment and are evaluated at the level of the individual. The last model expressed Communication as a function of the level of self-reported team experience frequency (low, moderate, or high), feedback privacy (Public or Private), trial (1–3), and a random effect of the team to which each participant belonged. The main effects for all models were evaluated by way of the estimated marginal means, calculated using the emmeans package in RStudio (Lenth, 2019).

When creating the LMM for Targeter Goal Awareness, the model was determined to be singular, meaning the variances of at least one linear combination of effects were zero or close to it. By examining the model, the standard error of the random effect was found to be zero. According to Bolker and Lüdecke (2019), dropping the random effect from the model in the case of zero-estimated variance components will not affect the estimated quantities, so a linear model was used rather than an LMM.

For Hypothesis 3, about the role switch’s impact, four *t*-tests, either independent or paired samples, were run to test four specific predictions. Prediction (a): Comparing Spotter performance in Trial 1 with the same Spotter’s performance in Trial 4 (SSSS role pattern) will show a baseline effect of both role and task experience. Prediction (b): Comparing a “new” Spotter in Trial 1 to a “new” Spotter in Trial 4 (who just switched to that role) will reveal an effect of task experience without role experience that increases communication. Prediction (c): A similar analysis for the Targeter (Targeter Trial 1 to a newly appointed Targeter in Trial 4) will reveal a similar effect of only task experience increasing communication. Lastly, Prediction (d) suggests that experienced Spotters in Trial 4 would have higher communication performance than new Spotters in Trial 4, or that role experience will positively impact communication performance even when task experience is the same.

All analyses of the Hypothesis 3 predictions were collapsed across the two feedback conditions. These included (1) a within-subjects test of Trial 1 communication vs. Trial 4 communication for the Spotter who doesn’t switch, (2) a between-subjects test of Trial 1 communication for Spotters vs. Trial 4 communication for the Targeter-turned-Spotter, (3) a between-subjects test of Trial 1 communication for the Targeter vs. Trial 4 communication for the Spotter-turned-Targeter, and (4) a between-subjects test of Trial 4 communication for the Spotter vs. the Targeter-turned-Spotter. These comparisons, except for comparison 1, were chosen to evaluate the performance of individuals new to the role and new to the task versus individuals who are new to the role but not new to the task. Comparison 1 was chosen as a baseline for performance differences from Trial 1 to Trial 4. Because of these four comparisons’ specificity, the effects of team were determined to have a limited effect on individual communication performance, and LMMs were not necessary. Linear regression and *t*-tests were used instead.

For Hypothesis 5, about the impact of teammate familiarity on communication, it was appropriate to use an Analysis of Variance (ANOVA) because the evaluation occurred at the level of the team. This hypothesis was tested at the level of the team rather than the level of the individual because the explanatory variable of teammate familiarity was not independent for members of each team. Additionally, the variable necessarily requires at least a dyad (due to the nature of teammate familiarity), further supporting analysis at the team level.

Unless otherwise noted, Tukey’s Honest Significant Difference (HSD) was used for multiple comparisons in pairwise differences. Cohen’s *d* values were calculated for each pairwise difference to indicate the effect size as a function of the standard deviations of the groups being compared. Cohen (1988) indicated that, when interpreting effect sizes, 0.2 ≤ *d* < 0.5 showed a small effect, 0.5 ≤ *d* < 0.8 could be considered a medium-sized effect, and *d* ≥ 0.8 showed a large effect, an interpretation the authors adopt in the present work.

## Results

This section presents the data analysis results and a direct answer to each of the posed hypotheses. Interpretation of these results will follow in Discussion.

### H1: Does Feedback Privacy Impact Team SA?

As shown in Table 6, public vs. private feedback did not result in a statistically significant difference for Shared Role Awareness, Team Task Awareness, or Targeter Goal Awareness. Therefore, H1 (public feedback will result in higher team SA than private feedback) was not supported. This counters previous literature (Geister et al., 2006), which showed that feedback that was targeted at the whole team would positively impact team SA.

**TABLE 6:** Differences in team SA evaluated by EMMs of feedback privacy.

**Response variable**	**95% CI**		***Df***	***t***	***p***	**Cohen’s *d***
	**LL**	**UL**				
Shared Role Awareness	−4%	13%	35	1.05	0.30	0.29
Team Task Awareness	5%	21%	35	1.28	0.21	−0.28
Targeter Goal Awareness	5%	13%	99	0.91	0.37	−0.21

*95% CI refers to difference of public–private feedback.*

### H2: Does Task Experience Impact Communication?

**FIGURE 3:**
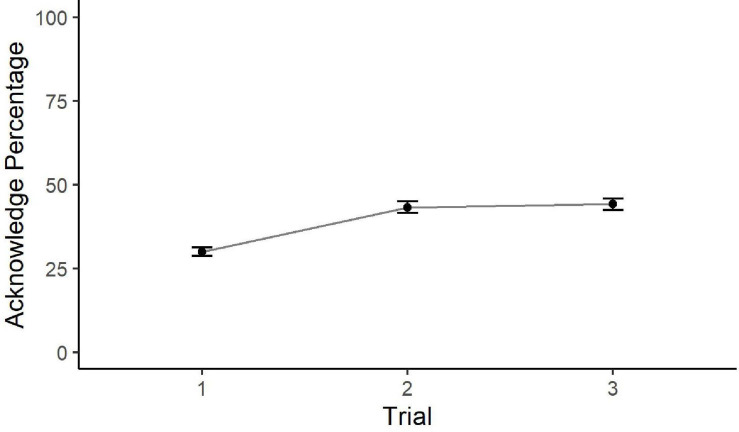
Mean Acknowledge Percentage and standard error by Trial. This hypothesis is based on an interest in quantifying the difference between the impact of task experience and the impact of role experience on communication. Results of the *t*-tests evaluating the four predictions of H3 are given in Table 7 and are further explained below.

Statistically significant differences in Acknowledge percentages were found between Trials 1 and 2, *t*(155) = −3.83, *p* < 0.001, *d* = −0.54, 95% CI [−20%, −6%], and Trials 1 and 3, *t*(156) = −4.12, *p* < 0.001, *d* = −0.59, 95% CI [−21%, −7%], but not between Trials 2 and 3, *t*(156) = −0.32, *p* = 0.95, *d* = −0.03, 95% CI [−8%, 6%]. Figure 3 shows the average change in Acknowledge percentage over Trials 1 through 3. Trial 1 Acknowledge percentage is significantly lower than Trials 2 and 3; however, Trial 2 communication performance is not significantly different from Trial 3, suggesting that no additional learning happens after Trial 2. Therefore, H2 (communication will improve as a participant gains experience in the task) was supported by the data.

### H3: Does Role Experience Impact Communication Beyond the Effects of Task Experience?

This hypothesis is based on an interest in quantifying the difference between the impact of task experience and the impact of role experience on communication. Results of the *t*-tests evaluating the four predictions of H3 are given in Table 7 and are further explained below.

**TABLE 7:** Differences in Acknowledge Percentage evaluated by independent and paired samples *t*-tests.

**Difference**	**95% CI**		***df***	***t***	***p***	**Cohen’s *d***
	**LL**	**UL**				
**Prediction a**						
SSSS T1–SSSS T4^§^	−14%	6%	25	−0.75	0.46	-0.13
**Prediction b**						
SSSS/T T1–TTTS T4	1%	26%	47	2.24	0.03*	0.61
**Prediction c**						
TTTS T1–SSST T4	−14%	35%	14	0.91	0.38	0.41
**Prediction d**						
SSSS T4–TTTS T4	1%	32%	49	2.18	0.03*	0.60

^§^Indicates a paired-samples *t-*test. All unmarked differences were evaluated with independent samples *t* tests. Significance at the *p* < 0.05 level is indicated by *.

**FIGURE 4:**
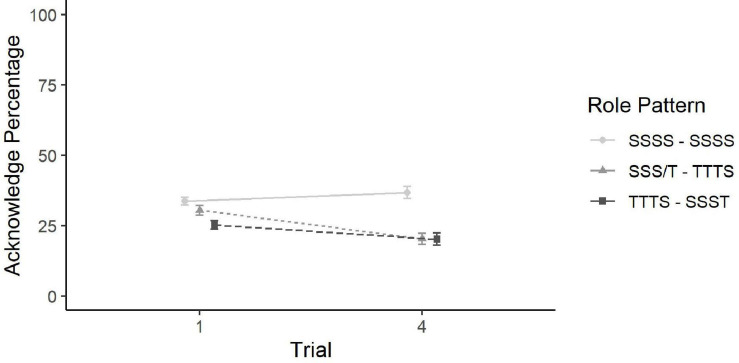
Line plot of the differences in Acknowledge Percentage over trial by Role Pattern. Error bars show standard error. Prediction (a) is shown by the first line with circular endpoints, Prediction (b) by the second line with the triangular endpoints, and Prediction (c) by the third line with the square endpoints. To interpret Prediction (d), compare the Trial 4 endpoints for predictions a and b.

For Prediction (a), spotters who remain in their role while also gaining task experience (role pattern SSSS), there was no significant difference in communication performance between Trial 1 (*M* = 34%, *SD* = 18%) and Trial 4 (*M* = 37%, *SD* = 29%) based on a paired samples *t*-test. For Prediction (b), there was a significant increase in communication performance between Spotters with no task and role experience (SSSS and SSST, Trial 1; *M* = 34%, *SD* = 18%) and Spotters with task, but not role experience (TTTS, Trial 4; *M* = 20%, *SD* = 25%) based on an independent samples *t*-test. The effect, based on Cohen’s *d*, was medium-sized. Next, for Prediction (c), data were analyzed using an independent samples *t*-test. There was no significant difference between Targeters with no task or role experience (TTTS, Trial 1; *M* = 30%, *SD* = 22%) and Targeters with task, but not role experience (SSST, Trial 4; *M* = 20%, *SD* = 29%). These differences are visualized by the lines in Figure 4.

Lastly, for Prediction (d), Trial 4 Spotters with role experience (SSSS) had significantly higher communication performance (*M* = 37%, *SD* = 29%) than Trial 4 Spotters without role experience (TTTS; *M* = 20%, *SD* = 25%), based on an independent samples *t*-test. The effect, based on Cohen’s *d*, was medium-sized, and the difference can be interpreted from Figure 4 via the difference between the Trial 4 circular endpoint and the Trial 4 triangular endpoint.

### H4: Does Experiencing a New Role Impact Team SA?

As detailed in Table 8, no statistically significant difference was found for Team Task Awareness, Shared Role Awareness, or Targeter Goal Awareness, between participants who did not switch roles in Trial 4 and those who did. Therefore, H4 (for the participants who experience two roles, team SA will be higher than that of participants who experience only one role) was not supported. This appears to counter previous research that found a team member’s experience in other roles (Volpe et al., 2006; Gorman et al., 2007).

**TABLE 8:** Pairwise differences of role pattern evaluated by EMMs of measures of team SA.

**Role Pattern Contrast**	**95% CI**		***df***	***t***	***p***	**Cohen’s *d***
	**LL**	**UL**				
**Team Task Awareness**						
SSSS–SSST	−10%	14%	66	0.35	0.93	0.09
SSSS–TTTS	9%	16%	67	0.64	0.80	0.21
SSST–TTTS	−11%	14%	68	0.29	0.96	0.10
**Shared Role Awareness**						
SSSS–SSST	−3%	4%	65	0.32	0.95	0.04
SSSS–TTTS	−1%	6%	65	1.34	0.38	0.18
SSST–TTTS	−1%	6%	65	1.02	0.57	0.15
**Targeter Goal Awareness**						
SSSS–SSST	−19%	4%	99	−1.32	0.39	−0.21
SSSS–TTTS	−19%	4%	99	−1.28	0.41	0.31
SSST–TTTS	−11%	12%	99	0.03	1.00	0.09

### H5: Does Teammate Familiarity Impact the Ability to Communicate?

Linear regression (and thereby, ANOVA) was used to evaluate the data for this hypothesis, as explained in section *“*Data Analysis*.”* Teams with no prior familiarity did not communicate significantly differently than teams with familiar members, *F*(1,54) = 0.28, *p* = 0.60, *d* = −0.14, 95% CI [−8%, 14%]. Therefore, H5 (teams with members who have prior familiarity will have better communication than those whose members do not) was not supported. This finding seems to counter previous research findings that familiarity improved team effectiveness in part by facilitating communication (Carpenter et al., 2008; Tong et al., 2013; Smith-Jentsch et al., 2015).

### H6: Does Teamwork Experience Impact Team SA?

As shown in Table 9, none of the differences in team SA performance based on prior teamwork experience were significant. Therefore, H6 (persons who have greater experience working in teams will have higher team SA) was not supported. This finding seems to counter previous research that found that team familiarity positively impacted performance (Huckman and Staats, 2011); however, it is worth noting that this result looks explicitly at just one aspect of team function and that familiarity, here, has to do with experience across a broad swath of teams, rather than consistent experience within a single team.

**TABLE 9:** Pairwise differences of teamwork experience evaluated by EMMs of measures of team SA.

**Teamwork experience contrast**	**95% CI**		***df***	***t***	***p***	**Cohen’s *d***
	**LL**	**UL**				
**Team Task Awareness**						
High–Low	−6%	6%	94	0.06	1.00	0.17
High–Moderate	−3%	7%	91	0.90	0.64	0.04
Low–Moderate	−3%	6%	87	0.85	0.67	0.29
**Shared Role Awareness**						
High–Low	−15%	19%	71	0.24	0.97	0.14
High–Moderate	−13%	15%	70	0.17	0.98	0.04
Low–Moderate	−15%	13%	69	−0.12	0.99	−0.10
**Targeter Goal Awareness**						
High–Low	−13%	16%	64	−0.12	0.99	−0.09
High–Moderate	−5%	19%	61	0.83	0.83	0.14
Low–Moderate	−6%	18%	54	0.80	0.71	0.24

## Discussion

The data did not support H1, which also counters previous literature that suggested that team-level feedback would result in more shared information than individualized feedback (Geister et al., 2006), thereby positively impacting the team’s SA. It could be that benefits related to shared information only occur after long exposures to the feedback, but more research is needed.

Alternatively, while this study was designed to reduce feedback overload in the public feedback condition by excluding Acknowledge task feedback, the public condition trials had more feedback messages (*M* = 11) than private condition trials (*M* = 9). This difference meant that participants received feedback every 27 s for the public feedback condition, while private condition participants received a feedback message every 33 s, on average. Also, no feedback was given for the Acknowledgment subtask in the public condition, which would give an incomplete picture of oneself and one’s teammates’ performance. Because of the ambiguity of public feedback and the more frequent disruptions, any potential increased team cognition may have been negated, and the feedback may have instead elicited a higher mental workload in the participants, negatively affecting their performance.

Indeed, participants often reported that the feedback was “*hard to pay attention to [while also attending to the duties of their role]*,” or “*it wasn’t really helpful and was difficult to understand*.” Some participants admitted to outright ignoring the feedback, and 37 of the 105 participants who gave comments reported that it was at least a little distracting. This perception was the same in both conditions, indicating that there was no relationship between the feedback’s helpfulness and the type of feedback received. Only two of the teams reported no problems using the feedback.

Per H2, participants were expected to perform better on communication as they gained experience in the task. From Trial 1 to Trial 2, participants improved in their ability to Acknowledge the communication of their teammates promptly; however, learning did not take place from Trial 2 to Trial 3. This is consistent with previous research on this task, that workload decreases from Trial 1 to 2 (indicating a learning effect), but not afterward (Ouverson et al., 2018).

In Trial 4, the role switch was used to examine whether someone with experience in the task, but not in their role, would have better communication than a participant with no task or role experience. This was statistically examined following four predictions of communication performance: establishing a baseline of performance using one role pattern (Prediction a), comparing two new Spotters (SSSS/SSST in Trial 1 to TTTS in Trial 4; Prediction b), comparing two new Targeters (TTTS in Trial 1 to SSST in Trial 4; Prediction c), and comparing the effects of role experience (Trial 4: SSSS to TTTS; Prediction d).

While it was expected that prior task experience (e.g., Trial number) would positively impact communication more than role pattern (and relatedly, the amount of role experience, as dictated by that pattern), it was found that role experience positively impacted communication (Prediction d), and task experience had no effect (Prediction a). It is expected that the lack of effect due to task experience is because the task requirements change with the roles in Trial 4, so any learned communication ability is effectively negated. Differences between role experience levels (Predictions b and c) were not consistently significant, as only prediction b showed significance. The difference between new Targeters but not new Spotters may suggest that the roles are not equally challenging. Since in Trial 4 we can compare only the two Spotters, due to a lack of a second Targeter, we cannot know if the difference between the outcomes of predictions b and c was because of a difficulty difference in the roles. For example, if we could compare new and old Targeters, we could determine if the role was harder to accomplish by the differences in communication performance between old Targeters and new Targeters.

In comparing communication performance for Spotters with equal amounts of task experience (Prediction d), those with higher levels of role experience had significantly higher communication performance. This difference is expected to be an artifact of the role switch in that our participants needed to adjust to their new role requirements. This finding does not necessarily counter the idea that Targeter role was more challenging, as the new Spotter could be committing more mental resources to the overall task out of habit and thereby neglecting the communication subtask even after switching to the more manageable task.

Beyond immediate impacts on communication, the act of experiencing another role was expected to impact team SA positively. The data did not support this hypothesis. One possible explanation for this is due to a limitation with the team SA measures. Scores on the team SA measures were also quite low, signifying that either the task was hard to understand or the measures were not adequately discriminatory. Another possibility is related to the workload induced by the experiment. Many participants reported that it was difficult to keep up with the tasks required of them. Changes in communication due to role experience were counter to expectations, as disruptions in requirements due to a change of role have more of a negative impact on performance than was expected.

Teams with members who knew each other before their involvement with the study were expected to communicate better than those without member familiarity. There was no difference in Communication dependent on familiarity. While a difference was expected, as familiarity had been shown to decrease the need for Communication while also positively impacting performance in previous research (Carpenter et al., 2008; Tong et al., 2013; Smith-Jentsch et al., 2015), two explanations for the lack of effect are possible. It is possible that (1) the presence of any familiarity is less important than the particular kind or length of familiarity when it comes to its effect on teamwork or (2) familiarity with team members is less important than an individual’s familiarity with teamwork in the particular context, in this case, a video game environment with solely verbal communication.

One notable limitation is that this experiment does not feature a feedback-free control condition. A control condition was not included in an effort to decrease the number of required participants. Because a control condition was not included in this iteration of testing, the tutor effectiveness (i.e., whether or not the tutor improved performance better than regular practice within the scenario) cannot be accurately evaluated. However, the analysis presented in Ostrander et al. (2019) shows that for a nearly identical two-person scenario and tutor framework combination, the presence of the ITTS resulted in a behavioral change of the team members. From this result, it is assumed that the feedback had some effect over just practice in the scenario.

## Conclusion and Future Directions

This paper conducts an initial exploration of how team skills are affected by the presence of an ITTS, as well as individual teammate differences in team member familiarity and both face-to-face and virtual team experience. While many of the hypothesized predictions have not garnered support, the authors present the following design and research objectives for future ITTSs and related research:

### Use Backup Behavior to Make Feedback Adaptive

Future work should be conducted to explore questions revealed in this study. The first consideration for future team tutoring studies is the expectation of backup behavior when offering feedback for actions and calculating team and team member performance. Backup behavior is the taking over of tasks for teammates when they are in need (McIntyre and Salas, 1995; Burke et al., 2015). Due to the complexity of attributing them correctly, backup behavior actions were counted as errors in the TESTT and resulted in feedback discouraging such actions. In the future, conditionals could be created to mitigate this by, for example, counting errors only when both actions in an interdependent task are missed. Additionally, biometric data, such as electrodermal activity (EDA), could be used to triangulate moments of need and modify the tutor’s understanding of learner behavior. Ideally, feedback would also adapt to members’ individual differences with team experience, providing additional scaffolding when necessary.

### Aid Adaptive Training by Matching Tasks to the Most Effective Feedback

More understanding of the types of feedback that are most appropriate for each variety of team task may be needed to shed light in this area. Some work has already been done to create a relevant taxonomy of the subtasks required of teamwork (Bonner et al., 2014), and similar efforts have explored the actions that aid workspace awareness (Gutwin and Greenberg, 2000, 2002). Future work that incorporates information about teamwork mechanics may be used to better adapt feedback to each subtask being evaluated. The training people received before conducting the task could be varied; for instance, the amount of training on their own task and the amount of cross-training on the other tasks may have an effect on team Cognition.

### Create More Robust Measures of SA

It would be worthwhile to consider backup behavior when outlining the team task’s expectations to improve the development of SA-evaluation tools, especially for tasks with a fast pace and unequal task distributions. Additionally, team SA should be measured repeatedly in the experiment rather than just at the end in order to understand how team SA changes over time. With robust measures of SA and repeated measurement, tutor performance can be better evaluated for its impact on team Cognition. Team performance on tasks gives an understanding of technical knowledge gained, but by knowing how a team’s understanding of the task and their teammates’ environments changes over time, their teamwork knowledge base can be evaluated.

### Establish a Balance Between Feedback Amount and Frequency of Messages

The present study of teamwork performance improvements included only a combined 20 min of exposure to the task and feedback. While this allowed an understanding of the impacts of feedback on an acute scale, it does not address the long-term effects or any transfer between task domains. It is also possible that 20 min of feedback exposure (or an average of 10 feedback messages) is not enough to impact performance.

When considering the amount of feedback given to learners, there is a lot of discussion around the impacts of “excessive messages” (see Walton et al., 2014; Gilbert S. B. et al., 2015; Ouverson et al., 2018; Price et al., 2018; Sinatra et al., 2018). The present study may offer additional insight into the correlated volume of messages that begins to have a negative effect. Future research could further evaluate the ideal frequency of feedback messages, perhaps by exploring the impact of filtering the feedback by testing differences in performance under various feedback filtering schemes, including the absence of filtering. This number may change depending on the number of individuals being simultaneously tutored while engaging in task-based conversation, and the adaptation of feedback amount based on the moment’s SA demands may be a new direction for feedback filtering.

On the other side of this balance, the reliance of ITTSs on simulated environments and serious games, as in the present study, suggests that more work to understand and develop the environmental cues to support awareness of the gamespace (the game-specific workspace in which gameplay and related activity occurs, see Wuertz et al., 2018) may remove unnecessary workload induced by the system. Especially when training real-life tasks in virtual environments, the user experience should be streamlined to allow maximum attentional focus on the task being trained. More research is needed to evaluate the impacts of user experience in simulator training and to understand the validity of training that incorporates game-based cues to help support team SA.

### Evaluate Communication Through Content in Addition to Timeliness

When considering effective team communication, performance likely depends on the timeliness of the communication, which was the focus of this study. The communication message’s content is likely more important than simply whether it occurs within a specific timeframe. Future research would do well to examine what is said and when it is said—a recommendation reiterated in direction 6.

### Use Natural Language Processing for Evaluation and Feedback Generation

While the analysis of keystrokes reveals some areas for further development in how the tutor evaluates user performance, more information could come from comparing the verbal utterances of the team members. For instance, comparing the performance of teams that use long phrases with those who are straight-to-the-point under various feedback conditions would give more information about the role of familiarity and communication style in teamwork performance. Similar to direction 5, natural language processing could be used to extract keywords and phrases from human speech, increasing the training’s efficacy. This could be useful in directing team onboarding efforts and could change how team tutoring begins in an ITTS by giving learners more information about their communication than is possible by only evaluating if it is present or absent.

### Contribution

Team training is becoming more virtual (both in delivery mechanisms and in being delivered to distributed team members) to keep pace with the changes in work and provide options for training for rare or dangerous events. As more studies are conducted to evaluate team training that utilizes an ITTS, this work will serve a foundational role in exploring the impact of an ITTS on team skill development. As the second iteration of a tutor developed using a scalable team surveillance task environment (STT to TESTT), this work showcases a platform that may be continually improved upon and used to develop and test team training.

## Data Availability Statement

The raw data supporting the conclusions of this article will be made available by the authors, without undue reservation.

## Ethics Statement

This study was reviewed and approved by the Iowa State University Institutional Review Board. Participants provided their informed consent to participate.

## Author Contributions

KO developed the experiment, ran subjects, analyzed the data, and wrote the manuscript as part of the requirements for her master’s degree. AO and JW ran subjects and contributed to data processing. AK helped extend the original simulated environment for use in this experiment, and contributed to data processing. MD, SG, EW, and AS contributed significant guidance during the planning, execution, and write-up of the experiment. All the authors contributed to the article and approved the submitted version.

## Conflict of Interest

The authors declare that the research was conducted in the absence of any commercial or financial relationships that could be construed as a potential conflict of interest.
